# Health care utilization in older people with cardiovascular disease in China

**DOI:** 10.1186/s12939-015-0190-y

**Published:** 2015-07-30

**Authors:** Lixia Dou, Xiaoyun Liu, Tuohong Zhang, Yangfeng Wu

**Affiliations:** School of Public Health, Peking University Health Science Center, No.38 Xueyuan Road, Beijing, China; China Center for Health Development Studies, Peking University Health Science Center, No.38 Xueyuan Road, Beijing, China; The George Institute for Global Health at Peking University Health Science Center, No.6 Zhichun Road, Beijing, China; Peking University Clinical Research Institute, Peking University Health Science Center, No.38 Xueyuan Road, Beijing, China

## Abstract

**Background:**

Population is ageing rapidly and prevalence of cardiovascular diseases is increasing in China. This study aims to examine the patterns of outpatient and inpatient health care utilization across different demographic and socioeconomic groups in older people with cardiovascular disease in China.

**Methods:**

Data were from World Health Organization (WHO) Study on Global Aging and Adult Health (SAGE) Wave 1. Chinese older people aged over 50 years with cardiovascular disease were included in the analysis. Outpatient and inpatient care utilization rates were presented and compared by demographic and socioeconomic characteristics. Multivariable logistic regression was used to examine the association between socioeconomic factors and health care utilization.

**Results:**

In total, 4162 older people with cardiovascular disease in SAGE China Wave 1 were included in the analysis. 86.4 % of them had health insurance. 54.9 % of the patients received outpatient care and 17.7 % received inpatient care over the past 12 months. Outpatient care utilization rate was significantly associated with age. Patients in older groups used more outpatient care than those in younger groups *(p = 0.010)*. Inpatient care utilization rate peaked at 70–79 years group (23.2 %), and then reduced to 17.5 % in 80 years plus group. Rich patients used more outpatient service than the poorer *(p < 0.001)*. No association was found between household wealth status and inpatient service utilization.

**Conclusion:**

Within the context of high health insurance coverage in China, the pattern of outpatient care utilization differs from that of inpatient care utilization among older patients aged over 50 years old with cardiovascular disease. Patients tend to use more outpatient care as they became older. As for inpatient care, the oldest patients aged over 80 years use less inpatient care than the 70–79 group. Household economic status plays an important role in outpatient care utilization, but it shows no association with inpatient care utilization in Chinese older patients.

## Background

Chinese population is ageing rapidly. The proportion of older people aged over 60 years reached 13.3 % in 2010 [1]. Burden of chronic diseases to the society is growing along with the aging population [2]. In particular, prevalence of cardiovascular disease, including hypertension, stroke and angina, is increasing, partially due to urbanization and lifestyle changes. In 2010, the prevalence of self-reported hypertension, angina and stroke has reached 26.7, 7.9 and 3.1 %, respectively, in Chinese older people aged 50 years and over [3]. The actual prevalence of cardiovascular disease in China might be even higher [3]. Rapid growth of ageing population and in the number of cardiovascular patients has brought great challenge to the health care system in China.

Health care utilization can be influenced by many factors including residence location, household economic status, and health insurance status [4]. Since the economic reform in early 1980s, China has witnessed increasing inequities in health wellbeing and health care utilization. People in low-income subgroups received less health care compared with their counterparts regarding their needs and the urban–rural disparity, especially in inpatient health care utilization, kept on widening [5]. Lack of health insurance was considered as a key barrier for people to access health services.

China’s health services are mainly delivered through three-tier hospital system, including primary health care (PHC) facilities, secondary hospitals and tertiary hospitals. Public hospitals are largely dependent on user fees as their main revenue source, with fee-for-service being the main provider payment method. In addition, there is no gatekeeping function at PHC level and patients can freely choose any public hospital for their first contact with PHC services. As a result, profit seeking behaviors, such as over prescription of medicines and medical examinations, are not uncommon in China’s health system [5]. In 2009, China launched an ambitious health system reform aiming at improving equity in health services utilization [6]. Since then health system in China has been undergoing radical changes with health insurance coverage being expanded rapidly [6]. It was expected that the increasing financial investment and expanding health insurance coverage will reduce the inequity in access to health service [7]. A study already reported that rural and urban disparity is shrinking in China [8].

Older people usually have relatively less income and therefore lower capacity to pay for their health service. In some cases this will prevent them from using health services [9]. The combination of high chronic diseases burden and limited income in old population indicates that their patterns of health care use might differ from those in younger population.

Understanding of the patterns of health care utilization in older patients is in urgent need. However, limited studies had been specifically designed to investigate this sub-population’s disease burden and health care behavior in China [3] and to our knowledge, even fewer studies focused on investigation of health service utilization in older patients with cardiovascular diseases. The World Health Organization (WHO) Study on global AGEing and adult health (SAGE) Wave 1 was designed and carried out to provide standardized data on health in older adults in six low- and middle-income countries. Our study aims to examine the patterns of outpatient and inpatient care utilization in older people with cardiovascular disease and the association of demographic and socioeconomic factors with health care utilization in China using data from WHO SAGE Wave 1 (China). Given the huge disease burden ahead of ageing Chinese population, our study will help identify gaps in health care utilization among older people across sub-groups and develop strategies to improve allocation of health resources efficiently and to promote equity in health care utilization in China.

## Methods

### Data sources

Public data from the WHO SAGE Wave 1 [10] were used in our study. WHO SAGE is a longitudinal study collecting data on health wellbeing and health care use of adults aged 50 years and older. SAGE has been conducted in China, Ghana, India, Mexico, South Africa and Russian Federation. The methodological details of SAGE have been described previously [3, 11, 12]. In brief, SAGE China Wave 1 was carried out during 2008 to 2010 using a multi-stage, stratified, random cluster sampling design. It is served as baseline data for a national sample of Chinese people aged 50 years and older. Trained interviewers carried out face-to-face interview with the eligible participants to collect information on demographics (including age and gender), socio-economic status (including location, education, household wealth and health insurance status) using standardized survey instruments. In total, SAGE China Wave 1 interviewed 13,177 respondents aged 50 years and older. The overall response rate was 92.3 %. For this study, we only include people aged 50 years and plus with self-reported cardiovascular diseases in the analyses.

### Inclusion criteria and definitions

Information on cardiovascular diseases was based on self-reported data. Respondents in SAGE China Wave 1 reported being diagnosed with hypertension, stroke and angina were included in our analyses.

Demographic and socioeconomic variables included age, gender, residence location (rural/urban), household wealth (quintiles) and health insurance status (yes/no). Age was grouped into four categories: 50–59 years, 60–69 years, 70–79 years and 80 years plus. The household wealth quintiles were based on possession of a set of household assets and a number of dwelling characteristics with Q_1_ representing the poorest household category and Q_5_ representing the richest household category [3].

Inpatient health care use was defined as at least one overnight stay in a hospital or other health care facility in past 12 months. Outpatient health care use was defined as any health care visit in the past 12 months except for overnight hospital stays.

### Statistical analysis

Statistical analysis was performed using SPSS 20.0. Outpatient and inpatient care utilization rates were calculated and compared between different demographic and socioeconomic groups. Gender, age, education, household wealth, residence location, and health insurance status were analyzed as explanatory variables. Comparisons were made using *χ*^2^ statistic for categorical variables and trend *χ*^2^ statistic for ordinal variables. Multivariable logistic regression models were used to examine the associations of demographic and socioeconomic factors with health care use. Dummy variables of three types of cardiovascular diseases were added to the regression model as a proxy indication of disease severity. Odds ratios (OR) were reported with 95 % confidence intervals (CIs) were reported. Statistical significance was assessed at *P* < 0.05.

### Ethical approval

Ethical approval of the study was obtained from Ethical Review Committee of Chinese Center for Disease Control and Prevention and the WHO Ethical Review Committee. Informed consent was obtained from each respondent prior to the review. The public data we used were anonymized.

## Results

There were 4185 older people with self-reported cardiovascular disease in SAGE China Wave 1. Twenty three were excluded because of missing data on household wealth status. Hence, a total of 4162 older people were included in the analysis. Among them, there were 3517 hypertension patients, 1133 angina patients and 450 stroke patients. Table 1 shows the distribution of demographic and socioeconomic characteristics of the patients. Overall 7.6 % were aged 80 years old or over, while this proportion (13.3 %) was greater in the sub-population of patients with stroke. 43.8 % of the patients were female, whereas there were more male (54 %) than female (46 %) among the stroke patients. 61.3 % patients lived in urban areas. 86.4 % reported that they had certain types of health insurance.

**Table 1:** Distribution (%) of characteristics, overall and by disease type

	Overall	Hypertension	Angina	Stroke
Characteristics	(*n* = 4162)	(*n* = 3517)	(*n* = 1133)	(*n* = 450)
Age (years)				
50-59	30.2	30.2	24.7	17.3
60-69	33.0	32.8	33.6	33.1
70-79	29.3	29.5	32.5	36.2
80+	7.6	7.5	9.2	13.3
Education				
Illiterate	24.6	25.0	23.3	24.2
Primary school or less	34.5	33.9	35.0	33.8
Secondary school	20.1	20.5	20.0	20.0
High school or above	20.8	20.6	21.6	22.0
Gender				
Male	43.8	43.7	37.9	54.0
Female	56.2	56.3	62.1	46.0
Wealth quintile				
Poorest	18.0	17.5	18.1	19.1
Q_2_	17.9	17.1	20.7	18.7
Q_3_	20.5	20.2	23.9	20.0
Q_4_	22.2	22.6	20.3	23.6
Richest	21.4	22.7	17.0	18.7
Residency				
Urban	61.3	61.6	66.8	66.9
Rural	38.7	38.4	33.2	33.1
Health insurance				
Yes	86.4	86.6	83.8	86.2
No	13.6	13.4	16.2	13.8

**Fig. 1:**
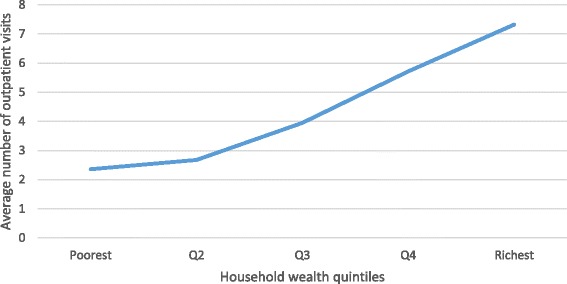
Average number of outpatient visits by household wealth quintiles

Table 2 shows the patterns of outpatient and inpatient health care utilization in these older people with cardiovascular disease. More than half (54.9 %) of the cardiovascular disease patients reported that they received outpatient health care over the past 12 months. As people getting older, they tended to use more outpatient care (52.4 % in 50–59 years group, 54.7 % in 60–69 years group, 56.5 % in 70–79 years group and 59.0 % in 80 years plus group; *p* = 0.010). Female patients had more outpatient service use than male patients (56.8 % versus 52.4 %, *p* = 0.005). Outpatient care utilization varied across household wealth groups. Rich patients were more likely to use outpatient care than the poorer (*p* < 0.001). Figure 1 shows that the average number of outpatient visits in the past 12 months was associated with household wealth status as well. Wealthy patients visited outpatient clinics more frequently than the poorer, with an average of 7.3 visits in the richest quintile and 2.4 visits in poorest quintile over the past 12 months. Patients who had insurance were more likely to use outpatient services than those who had no health insurance (57.0 % versus 41.4 %, *p* < 0.001). In regard to inpatient care, 17.7 % patients with cardiovascular disease received the service over the past 12 months. Inpatient service utilization rate increased from 14.2 % in 50–59 years group to 23.2 % in 70–79 years group, however, the inpatient service utilization rate in the 80 years plus group (17.5 %) was less than that in the 70–80 years group. There was no significant difference in inpatient care utilization between female and male patients (17.1 % versus 18.5 %, *p* = 0.246). Patients in different household wealth groups had similar level of inpatient care utilization. Patients with health insurance were more likely to use inpatient services than those without health insurance (18.4 % versus 13.4 %, *p* = 0.004).

**Table 2:** Outpatient and inpatient health care utilization by demographic and socioeconomic characteristics

	Patients	Outpatient care		Inpatient care	
	n	n(%)	p-value	n(%)	p-value
Total	4162	2284 (54.9 %)		737 (17.7 %)	
Age groups					
50-59	1256	658 (52.4 %)	0.010^*^	178 (14.2 %)	<0.001
60-69	1372	751 (54.7 %)		221 (16.1 %)	
70-79	1219	689 (56.5 %)		283 (23.2 %)	
80+	315	186 (59.0 %)		55 (17.5 %)	
Education					
Illiterate	1025	560 (54.6 %)	0.001	195 (19.0 %)	0.423
Primary school	1437	741 (51.6 %)		237 (16.5 %)	
Secondary school	836	460 (55.0 %)		152 (18.2 %)	
High school or above	864	523 (60.5 %)		153 (17.7 %)	
Gender					
Male	1823	956 (52.4 %)	0.005	337 (18.5 %)	0.246
Female	2339	1328 (56.8 %)		400 (17.1 %)	
Household wealth					
Poorest	748	333 (44.5 %)	<0.001^*^	133 (17.8 %)	0.700
Q_2_	743	360 (48.5 %)		120 (16.2 %)	
Q_3_	855	450 (52.6 %)		159 (18.6 %)	
Q_4_	925	577 (62.4 %)		171 (18.5 %)	
Richest	891	564 (63.3 %)		154 (17.3 %)	
Location					
Urban	2551	1434 (56.2 %)	0.029	456 (17.9 %)	0.722
Rural	1611	850 (52.8 %)		281 (17.4 %)	
Health insurance					
Yes	3595	2049 (57.0 %)	<0.001	661 (18.4 %)	0.004
No	567	235 (41.4 %)		76 (13.4 %)	

^*^Trend *χ* ^2^ test

Table 3 shows results of multivariable logistic regression of potential factors associated with outpatient and inpatient care utilization. Age was associated with outpatient care utilization. Compared with 50–59 years group, 70–79 years group (OR: 1.26, 95 % CI: 1.06-1.51) and 80 years plus group (OR: 1.38, 95 % CI: 1.05-1.80) used more outpatient care. Female patients used more outpatient service than male patients (OR: 1.30, 95 % CI: 1.13-1.48). Patients in the richest quintile used more outpatient service than those in the poorest quintile (OR: 2.01, 95 % CI: 1.61-2.51). Health insurance status was associated with outpatient care utilization and patient with health insurance used more service than those without health insurance (OR: 1.67, 95 % CI: 1.37-2.03). No association was found between education and outpatient care utilization. In regard to inpatient care, the patients in 70–79 years group used more services than those in 50–59 years group (OR: 1.65, 95 % CI: 1.32-2.08), whereas no significant difference in utilization was found between 80 years plus group and 50–59 years group (OR: 1.03, 95 % CI: 0.72-1.47). Patients with health insurance used more service than those without health insurance (OR: 1.51, 95 % CI: 1.14-1.99). Household wealth status and education were not associated with inpatient care utilization.

**Table 3:** Logistic regression of factors associated with outpatient and inpatient care utilization

	Outpatient care		Inpatient care	
	OR (95 % CI)	p-value	OR (95 % CI)	p-value
Age groups				
50-59	1	--	1	--
60-69	1.16 (0.99-1.36)	0.073	1.09 (0.87-1.36)	0.458
70-79	1.26 (1.06-1.51)	0.008	1.65 (1.32-2.08)	<0.001
80+	1.38 (1.05-1.80)	0.019	1.03 (0.72-1.47)	0.867
Education				
Illiterate	1	--	1	--
Primary school	0.88 (0.74-1.05)	0.165	0.86 (0.68-1.08)	0.195
Secondary school	0.96 (0.77-1.20)	0.741	1.02 (0.77-1.35)	0.885
High school or above	1.06 (0.84-1.33)	0.627	0.89 (0.66-1.20)	0.432
Gender				
Male	1	--	1	--
Female	1.30 (1.13-1.48)	<0.001	0.91 (0.77-1.08)	0.290
Household wealth				
Poorest	1	--	1	--
Q_2_	1.16 (0.94-1.42)	0.177	0.86 (0.65-1.14)	0.305
Q_3_	1.34 (1.09-1.65)	0.006	1.04 (0.79-1.36)	0.802
Q_4_	1.95 (1.58-2.41)	<0.001	1.06 (0.81-1.40)	0.675
Richest	2.01 (1.61-2.51)	<0.001	1.04 (0.78-1.39)	0.777
Location				
Urban	1	--	1	--
Rural	1.00 (0.85-1.17)	0.955	1.08 (0.88-1.33)	0.479
Health insurance				
Yes	1	--	1	--
No	1.67 (1.37-2.03)	<0.001	1.51 (1.14-1.99)	0.004
Hypertension				
No	1	--	1	--
Yes	1.40 (1.14-1.72)	0.001	1.22 (0.96-1.55)	0.098
Angina				
No	1	--	1	--
Yes	1.05 (0.89-1.24)	0.558	2.07 (1.70-2.51)	<0.001
Stroke				
No	1	--	1	--
Yes	1.50 (1.21-1.86)	<0.001	2.51 (1.99-3.16)	<0.001

## Discussion

Using data from SAGE China Wave 1, this paper analyzed the patterns of health care utilization among 4162 older people aged 50 and over with self-reported cardiovascular diseases. We found that health care utilization was relatively low in this population in China. Determinants of outpatient care utilization were not the same as those of inpatient care utilization. Age, gender, household wealth status and health insurance were associated with the outpatient care utilization, while only age and health insurance status had an association with the inpatient care utilization. Within the current context of high health insurance coverage in China, inequity was more evident in outpatient care utilization than that in inpatient care utilization. Our study adds considerable knowledge to current understanding of health care utilization patterns among older people with cardiovascular diseases in China, with respect to the range of health care services and the association with demographic and socioeconomic factors.

In China, the overall levels of health care utilization were generally low in older patients with cardiovascular diseases. The Chinese guidelines for the management of hypertension recommend that patients with hypertension should visit their physicians at least once every 1 to 3 months and patients with uncontrolled hypertension should increase the frequency of physician visiting [13]. Other clinical guidelines also require closely monitoring of blood pressure and treatment of hypertension, along with management of serum lipid and treatment of diabetes, for patients with stroke/angina in secondary prevention of the diseases [14, 15]. Clinical treatment of cardiovascular diseases is long-term and expensive, hence inequity of health care utilization is not uncommon in many countries, particularly in developing countries [16, 17]. In our study nearly half of the patients reported no use of outpatient service in the past year. Inpatient utilization rate was less than 18 % over the past 12 months. Underuse of health care service, particularly outpatient service, by patients with chronic diseases may cause poor adherence to disease management protocol and hence increase hospitalization and health care cost.

In our study we found that the pattern of outpatient care utilization was different from that of inpatient care utilization across different age groups in older people with cardiovascular disease in China. Research undertaken in developed countries shows that ill health is becoming more and more compressed into later years and health care utilization tend to peak at about 80 years of age and over [18, 19]. However, another research reported that unlike developed countries, general health care utilization, including both inpatient and outpatient service, peaked at the sub-group of people aged 70 to 79 years in low- and middle-income countries [4]. Our findings tell a different story and in China patients tended to use more outpatient care as they grew older with the service utilization peaking at the group of patients aged 80 years and plus. Whereas in regard to inpatient care, the utilization pattern was similar to that in other developing countries [4] and it peaked at 70–79 years age group and then declined in the 80 years and over group. This might be influenced by patients’ willingness to receive services [20]. Further research is needed to explore the issue.

Health care utilization patterns differed in men and women. Previous studies found that women were more likely to use preventive and diagnostic services compared with men [21, 22], though men tended to have a higher mortality in late-life [23, 24]. In regard to outpatient care, our study also found that older women with cardiovascular diseases used more services than older men. This result is consistent with other analyses in general older population [4, 22]. However, evidence of gender differences in the utilization of inpatient health are contradictory [4, 22, 25, 26]. Some studies reported that women made less use of emergency and inpatient services than men [4, 22, 26], while others reported no statistical differences in hospital admissions between men and women [25]. In our study we observed no significant inequity in inpatient service use between men and women in the sub-population of older population with cardiovascular diseases in China.

Socioeconomic status played an important role in outpatient care utilization, but had no significant association with inpatient care utilization in China. Previous studies suggested that wealthier people tended to use more health service [26, 27]. Socioeconomic status was inversely associated with ill health but positively associated with hospitalization, even after controlling for health status, age and social support [26]. In our study we also found that inequity was indicated by the positive association between household wealth and outpatient service use. Both the likelihood and frequency of outpatient care utilization in wealthy older people were greater than that in the poorer, though all cardiovascular patients are in need of regular health care to monitor risk factors in secondary prevention. However, in contrast to previous studies, our study found no significant association of inpatient care utilization with household economic status. There was no evidence of pro-rich inequity in inpatient health care utilization in our analysis, perhaps due to the high coverage of health insurance [17] and Chinese culture of family support [28].

In recent years, health insurance coverage has been rapidly expanding in China. China has three main health insurance schemes, including new cooperative medical scheme for rural population, urban employment based basic health insurance and urban residents based basic health insurance. Health insurance scheme can remove financial barrier for patients to get access to health care and therefore release patients’ needs for health care utilization. In our study more than 85 % had participated at least one type of health insurance schemes. Health insurance status showed a strong association with health care utilization. Patients with health insurance were more likely to use health service than those without health insurance. However, insurance schemes may not adequately protect people against the long-term outpatient costs associated with chronic diseases [29]. This may explain why poorer group still used less outpatient care than the rich group in these older patients, even after controlling health insurance status and other potential confounding factors.

Our study has several limitations. First, the data was from a cross-sectional survey which does not allow for causal inference between potential risk factors and health care utilization in our study. Second, the diagnosis of cardiovascular diseases was based on self-reported data which are subject to underdiagnoses and misdiagnosis, since quality of diagnosis can vary dramatically between different health care settings. Third, different types of health insurance schemes differ in designs and contents of benefit packages in China and hence might affect patients’ health care utilization in various ways. However, in this study we did not have data on individual patients’ health insurance types and therefore were unable to measure the impact of different health insurance schemes on health care utilization. But it is still evident in our study that the pattern of outpatient care utilization differed from that of inpatient care utilization among patients with cardiovascular disease in China. Within the current context of high health insurance coverage in China, inequity in health care utilization, particularly outpatient care utilization, remains in older patients with cardiovascular disease. This issue needs to be further investigated and to be addressed in order to close the gaps in access to and utilization of health care across different groups.

## Conclusions

Within the current context of high health insurance coverage in China, health care utilization was still relatively low in older Chinese patients aged 50 and over with cardiovascular diseases. The pattern of outpatient care utilization differs from that of inpatient care utilization. Age is positively associated with outpatient care utilization, whereas in regard to inpatient care, the oldest patients aged over 80 years use limited inpatient services according to their needs. Women are more likely to use outpatient care than men, whereas men use similar, if not more, inpatient services as women. Household economic status plays an important role in outpatient care utilization, but had no significant association with inpatient care utilization. This may indicate inequity in outpatient health care utilization among different social economic groups of older patients with cardiovascular disease who are in need of regular health care. These findings have important policy implications on equity of health care utilization in Chinese older patients. Health insurance schemes need to be further reformed to prioritize outpatient care use. Special attention should be paid to the patients aged 80 years and over to promote their access to inpatient services. Further research is needed to investigate the causes behind the inequity in health care utilization in Chinese older patients.
